# Three-Dimensional SERS Substrates Formed with Plasmonic Core-Satellite Nanostructures

**DOI:** 10.1038/s41598-017-13577-9

**Published:** 2017-10-12

**Authors:** Li-An Wu, Wei-En Li, Ding-Zheng Lin, Yih-Fan Chen

**Affiliations:** 1 Institute of Biophotonics, National Yang-Ming University, Taipei, 112 Taiwan; 2 Material and Chemical Research Laboratory, Industrial Technology Research Institute, Hsinchu, 310 Taiwan; 3 Biophotonics and Molecular Imaging Research Centre, National Yang-Ming University, Taipei, 112 Taiwan

## Abstract

We demonstrate three-dimensional surface-enhanced Raman spectroscopy (SERS) substrates formed by accumulating plasmonic nanostructures that are synthesized using a DNA-assisted assembly method. We densely immobilize Au nanoparticles (AuNPs) on polymer beads to form core-satellite nanostructures for detecting molecules by SERS. The experimental parameters affecting the AuNP immobilization, including salt concentration and the number ratio of the AuNPs to the polymer beads, are tested to achieve a high density of the immobilized AuNPs. To create electromagnetic hot spots for sensitive SERS sensing, we add a Ag shell to the AuNPs to reduce the interparticle distance further, and we carefully adjust the thickness of the shell to optimize the SERS effects. In addition, to obtain sensitive and reproducible SERS results, instead of using the core-satellite nanostructures dispersed in solution directly, we prepare SERS substrates consisting of closely packed nanostructures by drying nanostructure-containing droplets on hydrophobic surfaces. The densely distributed small and well-controlled nanogaps on the accumulated nanostructures function as three-dimensional SERS hot spots. Our results show that the SERS spectra obtained using the substrates are much stronger and more reproducible than that obtained using the nanostructures dispersed in solution. Sensitive detection of melamine and sodium thiocyanate (NaSCN) are achieved using the SERS substrates.

## Introduction

Plasmonic nanostructures of various kinds of geometries have been synthesized and utilized for bio- and chemical sensing in recent years^1^. It has been known that localized surface plasmon resonance (LSPR) of noble metal nanoparticles can induce intense and highly localized electromagnetic fields for surface-enhanced Raman spectroscopy (SERS) and metal-enhanced fluorescence^2^. Since Raman signals can be acquired within seconds from a small amount of sample without complicated sample preparation procedures and can be analysed to provide a “fingerprint” of the molecule being observed, Raman spectroscopy has drawn much attention recently for its applications in food safety testing and environmental monitoring^3–9^. When Au and Ag nanoparticles (AuNPs and AgNPs) are used for SERS detection of molecules, the distance between adjacent nanoparticles should be as short as a few nanometres to obtain high Raman enhancement^10,11^. In addition to the interparticle distance, which can be fine-tuned to optimize the SERS effects^12–14^, the size, shape, composition and surrounding environment of metal nanoparticles should also be carefully controlled to achieve sensitive and reproducible SERS detection^2^. To assemble metal nanostructures that have precisely controlled geometry and many small gaps as SERS hot spots, various top-down lithographic approaches and bottom-up self-assembly methods have been developed recently. Molecular self-assembly is one of the bottom-up methods that can be used to assemble two- dimensional (2D) or three-dimensional (3D) nanostructures in a simple and cost-effective way^12,14–17^. While randomly aggregated AuNPs or AgNPs can already be used to enhance Raman signals, using DNA or other molecules to direct the assembly of nanoparticles allows us to have a better control of the geometries of nanoparticle-based SERS substrates and thus their SERS effects. Given that DNA molecules of different lengths and sequences can be synthesized easily, nanoparticles can be assembled into a designed nanostructure through DNA hybridization or attachment of end-labelled functional groups^18–23^. For example, it has been demonstrated that Au-Ag core-shell nanodumbbells^12,13^ and plasmonic core-satellite nanostructures can be assembled through DNA hybridization^14,24–28^ or binding of other linker molecules^29–34^. The SERS effects of the nanoparticle-based nanostructures can be optimized by controlling the gaps between adjacent nanoparticles through coating Ag shells around the nanoparticles^12–14^. In addition, when a DNA aptamer is used as a linker to attach two nanoparticles, an analyte-controllable SERS hot spot at the nanogap between the nanoparticles can be created^28,35–37^.

When using either metal nanoparticles or nanostructured substrates for SERS detection of molecules, it is important to have many electromagnetic hot spots at the focal point of the Raman excitation laser to obtain good sensitivity. While various kinds of SERS substrates have been developed in recent years, the hot spots of many of these substrates, which are made by either nanofabrication or self-assembly of nanoparticles, are usually limited to a thin 2D plane that has a thickness of a few tens of nanometres. Given that the focal volume of a Raman excitation laser is a 3D space rather than a 1D line or 2D plane, increasing the number of SERS hot spots in the z-direction allows for better use of the focal volume and thus can increase Raman signals^38–41^. In addition, a larger surface area of a 3D substrate allows more molecules to bind to the substrate, which is also beneficial for the sensitivity of detection.

In this study, to obtain a high density of SERS hot spots in all three dimensions, we used a DNA-assisted assembly method to immobilize AuNPs on the surfaces of micron-sized polymer beads to create core-satellite nanostructures that had many nanogaps between the AuNPs. To maximize the enhancement factor and the number of nanogaps, we investigated the experimental parameters that could increase the density of the immobilized AuNPs, and we fine-tuned the interparticle distance through adding a Ag shell to the AuNPs. In addition, since the nanostructures synthesized in this study were dispersible in solution rather than immobilized on a fixed substrate, we were able to prepare 3D SERS substrates by accumulating the core-satellite nanostructures using a drying process. Given that the size of the laser spot was much larger than the size of each core-satellite nanostructure, the number of SERS hot spots excited by the laser significantly increased after we accumulated the dispersed nanostructures.

The hot spots of our 3D SERS substrates, which were formed with plasmonic core-satellite nanostructures, were densely distributed not only on a 2D plane but also in the z-direction along the micron-sized bead surface. Although recent studies have demonstrated various kinds of core-satellite nanostructures, some of the studies only focus on the synthesis and plasmonic properties of the nanostructures but did not use the nanostructures for SERS^24,25,32,42–47^. Some other studies used core-satellite nanostructures for enhancing Raman reporter molecules that were placed at the hot spots but not for determining the concentrations of molecules^30,31,33,34,48–50^. Many of the core-satellite nanostructures used for SERS detection of molecules were based on detection schemes that utilized either DNA aptamers and Raman reporter molecules^28,29,36,37,51–53^ or certain kinds of chemical interactions^54–56^, but the availability of suitable aptamers and chemical reactions could limit the use of the nanostructures for the detection of other molecules. Some of the SERS substrates formed with core-satellite nanostructures did not have many SERS hot spots in the z-direction, and the density of the nanostructures in the xy-plane was not maximized^14,27,57^. In this study, we did not use any specific aptamer, Raman reporter, or chemical reactions for SERS detection. We determined the sensitivity and reproducibility of our SERS substrates by measuring SERS spectra of melamine, which was once found to be illegally added to milk products^58,59^, and sodium thiocyanate (NaSCN), which is a preservative for milk^60^ but could affect thyroid function when its concentration is too high^61^.

## Results and Discussion

### Synthesis of core-satellite nanostructures

**Figure 1:**
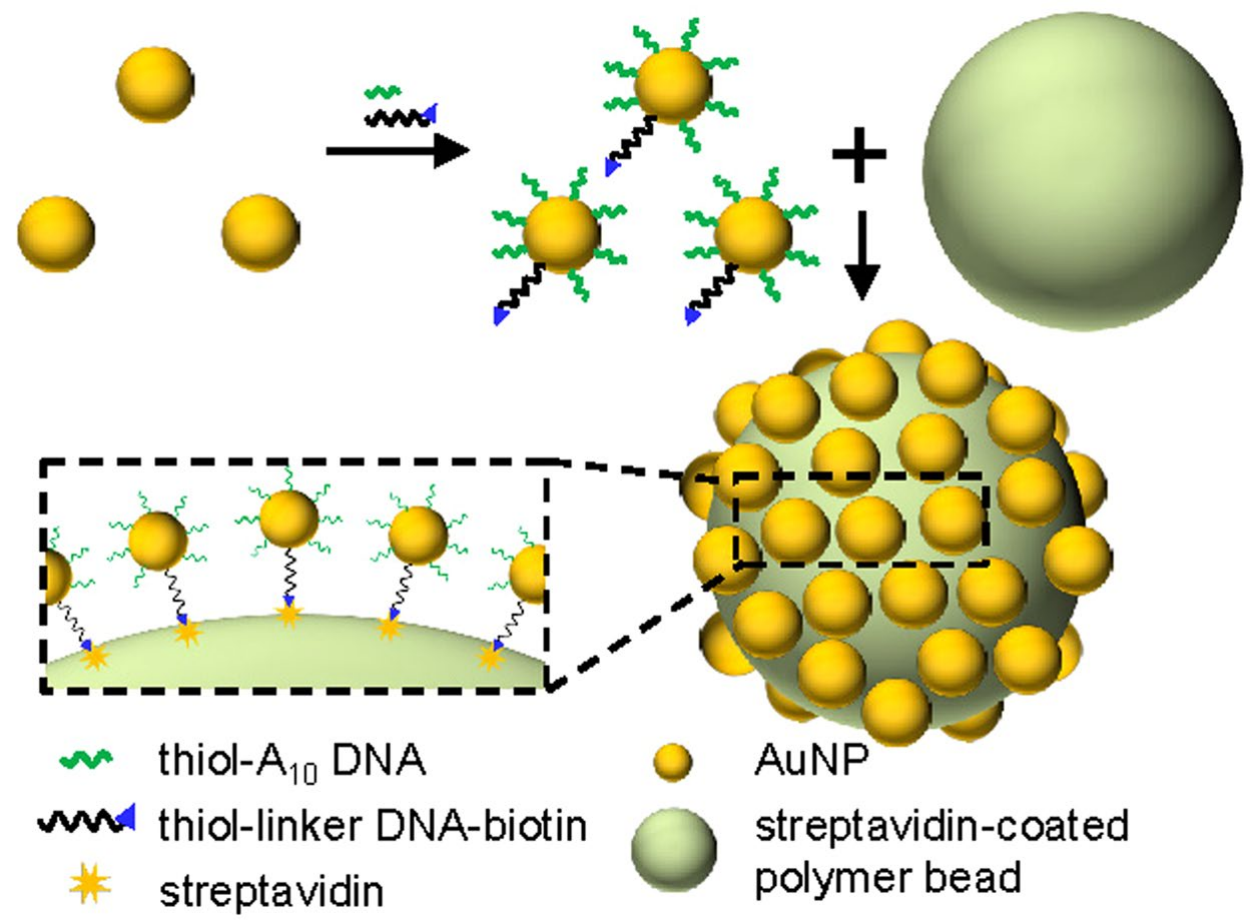
Schematic representation of the assembly process for the core-satellite nanostructure. The AuNPs were functionalized with two kinds of DNA for stabilization and attachment to the polymer bead through binding between streptavidin and biotin.

The schematic showing the assembly of the core-satellite nanostructure is shown in Fig. 1. To synthesize the nanostructure, the AuNPs were functionalized with thiol-modified A10 DNA molecules as well as dual-labelled linker DNA molecules, which were modified with thiol on one end and biotin on the other, using a method similar to that reported by Xu *et al*.^62^. Briefly, by using Tween 80 as a protective agent, the two kinds of DNA molecules were conjugated to the AuNPs under high salt concentration. Then, the AuNPs were immobilized on the surfaces of streptavidin-coated polymer beads through the binding between streptavidin and biotin to form the core-satellite nanostructures. It should be note that, while the biotin-labelled DNA is essential to the immobilization of the AuNPs, the number of the biotin-labelled DNA molecules immobilized on each AuNP should be low enough to avoid multiple polymer beads being attached to a AuNP and causing aggregation of the beads. On the other hand, since the AuNPs formed aggregations in a salt solution when the number of DNA molecules attached to each AuNP was too low, we functionalized the AuNPs with many thiol-modified A10 DNA molecules in addition to the biotin-labelled DNA to stabilize the AuNPs. To improve the SERS effects of the assembled core-satellite nanostructures, we optimized the parameters for the AuNP immobilization to immobilize a high density of the AuNPs on the polymer beads, and we fine-tuned the sizes of the nanogaps between the adjacent immobilized AuNPs by adding a Ag shell to the AuNPs, which will be detailed later.

**Figure 2:**
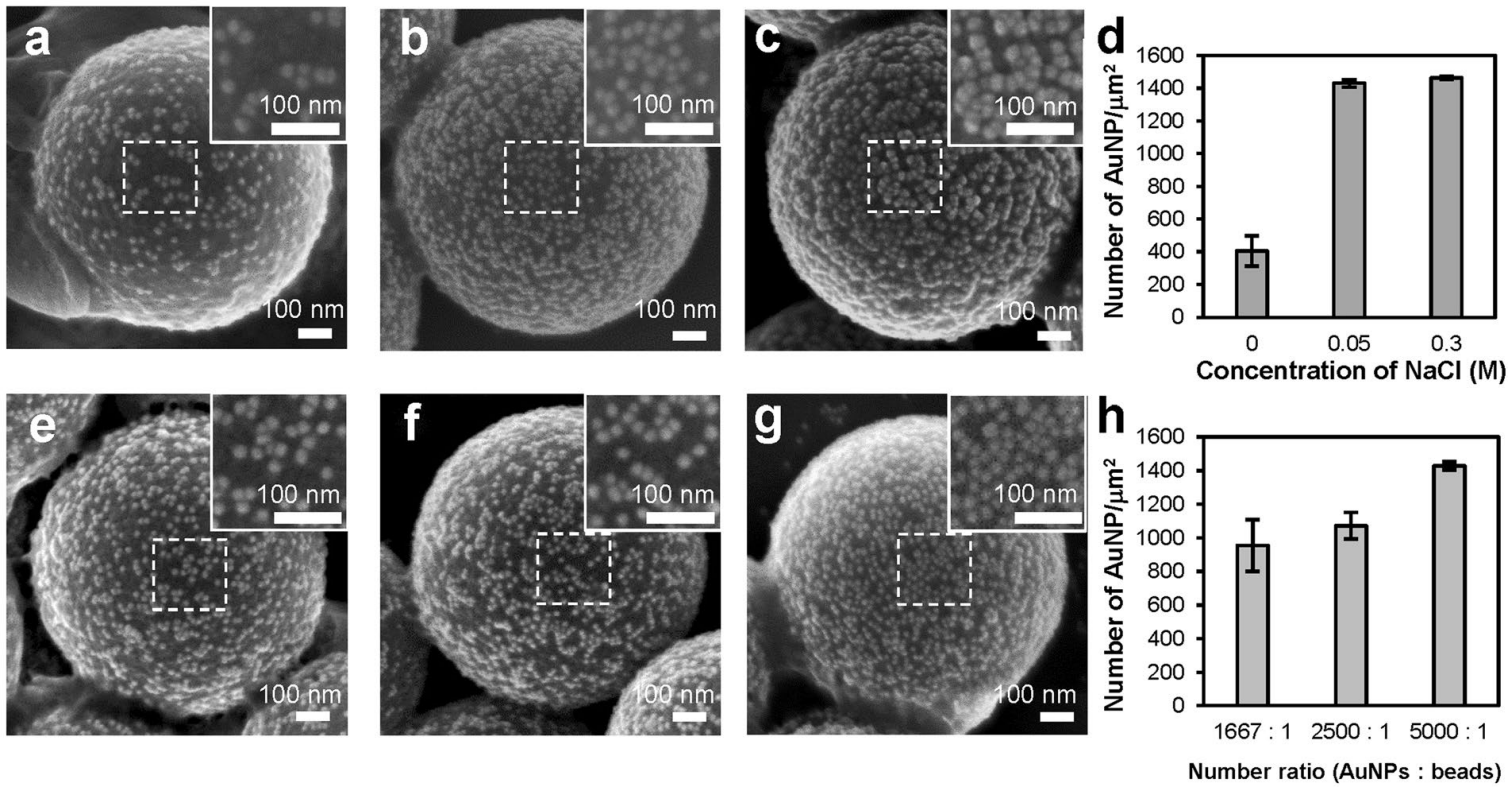
Effects of NaCl concentration and the number ratio of the AuNPs to the polymer beads on the AuNP immobilization. (**a**–**c**) Representative SEM images showing the spatial distribution of the AuNPs immobilized on the polymer beads at (**a**) 0 M, (**b**) 0.05 M, and (**c**) 0.3 M NaCl solution. (**d**,**h**) The densities of the AuNPs immobilized (d) at three different NaCl concentrations and (**h**) with three different number ratios of the AuNPs to the polymer beads (*n* = 10 per experimental condition). Error bars, SD. (**e**–**g**) Representative SEM images showing the core-satellite nanostructures assembled with three different number ratios of the AuNPs to the polymer beads: (**e**) 1667:1, (**f**) 2500:1, (**g**) 5000:1.

To increase the density of the AuNPs immobilized on each polymer bead as much as possible, we investigated the effects of salt concentration of solution and the number ratio of the AuNPs to the polymer bead on the AuNP immobilization. Figure 2a–c are the scanning electron microscope (SEM) images showing the distribution of the AuNPs immobilized at different NaCl concentrations. To quantify the results, for each condition we used three SEM images to calculate the number of the AuNPs in a square area (250 μm × 250 μm) located at the centre of each SEM image. As shown in Fig. 2d, the densities of the immobilized AuNPs were 405, 1429, and 1461 particles/μm^2^ for the core-satellite nanostructures assembled at 0 M, 0.05 M, 0.3 M NaCl solution, respectively. Figure 2a shows that, when there was no NaCl in solution, the AuNPs could not be densely immobilized because of the repulsive forces from the negatively charged DNA. On the other hand, as shown in Fig. 2b,c, the density of the immobilized AuNPs was significantly increased when the concentration of NaCl was raised to 0.05 M and 0.3 M. However, as shown in Fig. 2c, we found that the AuNPs formed small clusters on the surfaces of the polymer beads under 0.3 M NaCl solution. A possible reason for the cluster formation was that the number of the DNA molecules immobilized on each AuNP was not enough to stabilize the AuNP under such high salt concentration. Given that adjacent metal nanoparticles on a SERS substrate should be close but not touching each other to achieve optimal SERS effects, we immobilized the AuNPs on the polymer beads under 0.05 M NaCl solutions in the later experiments.

Figure 2e–g are the representative SEM images of the core-satellite nanostructures assembled with three different number ratios of the AuNPs to the polymer beads. As shown in Fig. 2e–g, the sizes of the nanogaps between adjacent nanoparticles decreased as the ratio changed from 1667:1 to 5000:1, and increasing the ratio further does not reduce the sizes of the nanogaps more. Based on the analysis of three SEM images for each condition, the densities of the immobilized AuNPs were 955, 1072, and 1429 particles/μm^2^ for the ratio of 1667:1, 2500:1, and 5000:1, respectively, as shown in Fig. 2h.

### Preparation of 3D SERS substrates

**Figure 3:**
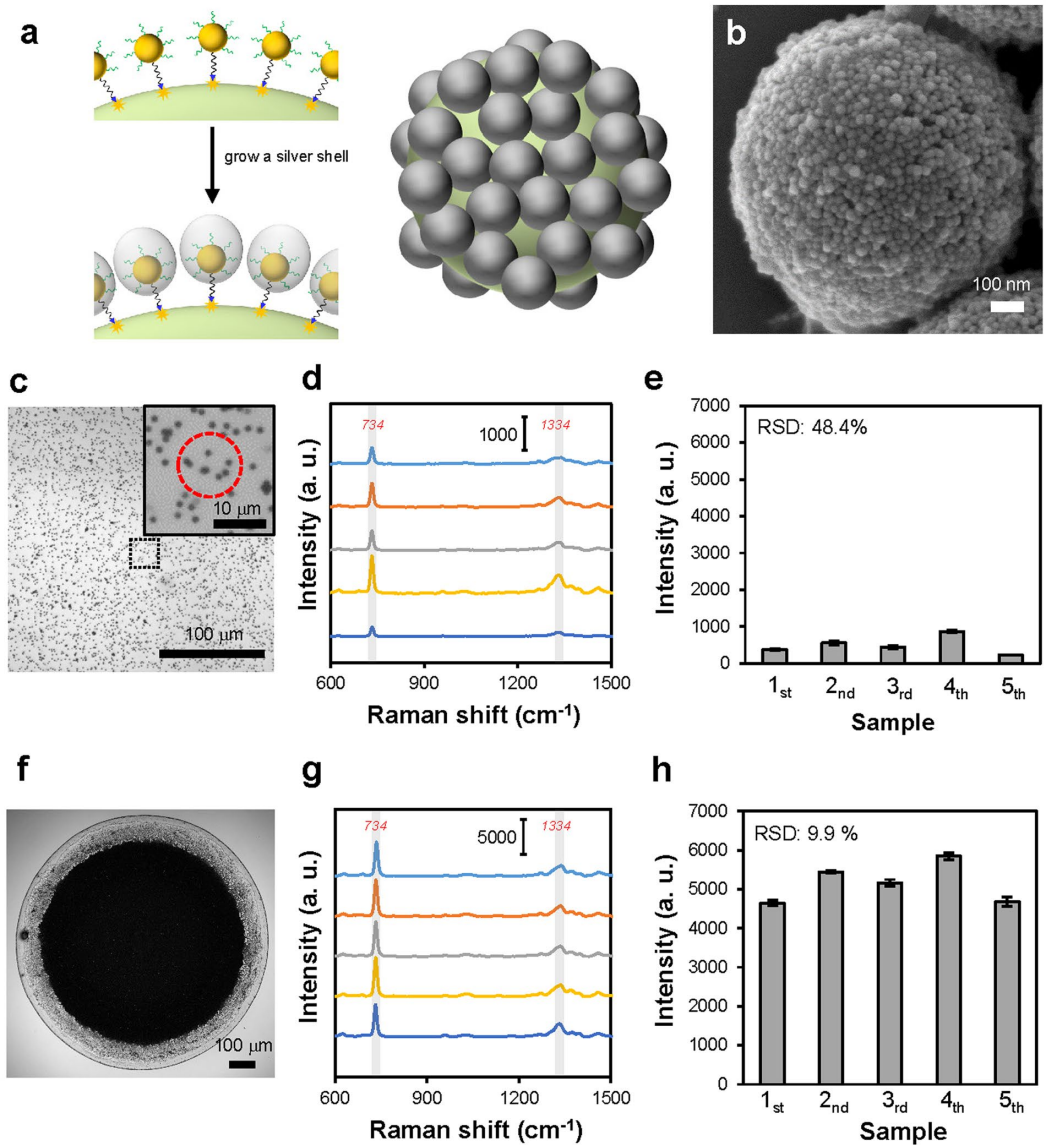
Addition of a Ag shell to the core-satellite nanostructure and utilization of the nanostructure in SERS measurements. (**a**) Schematic representation of the growth of a Ag shell on the AuNPs immobilized on the polymer beads. (**b**) Representative SEM image of the core-satellite nanostructure coated with a Ag shell. (**c**,**f**) Representative optical microscope images showing the core-satellite nanostructures (**c**) dispersed in solution and (**f**) accumulated by drying a nanostructure-containing droplet suspended on a PDMS sheet. When using substrates similar to that shown in (**f**) for SERS measurement, the Raman laser was focused at the central regions of the substrates. Inset in (**c**) is a zoom-in view of the dispersed nanostructures. The red dashed circle indicates the size of the laser spot. (**d**,**g**) SERS spectra of 100 μM adenine measured using (**d**) five mixtures containing the same batch of dispersed nanostructures and (**g**) five substrates similar to that shown in (**f**). (**e**) The intensities of the Raman peak of adenine at 734 cm^−1^ in the spectra shown in (**d**). (*n* = 10 per sample). Error bars, SD. (**h**) The intensities of the Raman peak of adenine at 734 cm^−1^ in the spectra shown in (**g**) (*n* = 10 per sample). Error bars, SD.

After immobilizing the AuNPs on the polymer beads to form the core-satellite nanostructures, we grew a Ag shell on the surfaces of the AuNPs, which then became Au-Ag core-shell nanoparticles, to reduce the interparticle distance further, as shown in Fig. 3a. The Ag shell was required for obtaining strong Raman enhancement since the gaps shown in Fig. 2g were still not small enough to serve as effective SERS hot spots^12^, though we had found the experimental conditions to densely immobilize the AuNPs. As shown in the SEM image (Fig. 3b), after the AuNPs of the nanostructure were coated with a Ag shell, the gaps between the nanoparticles indeed became smaller than that shown in Fig. 2g. The extinction spectra of the nanostructures in water before and after the addition of the Ag shell are shown in Supplementary Fig. S1.

Before we fine-tuned the thickness of the Ag shell to optimize the SERS effect, we compared two different methods to utilize the core-satellite nanostructures in SERS measurements. We used adenine as a model analyte to understand the intensity and reproducibility of the Raman signals obtained using the two methods. As shown in Fig. 3c, we first used the core-satellite nanostructures dispersed in solution to enhance the Raman signals of 100 μM adenine, and we compared the SERS spectra obtained from five mixtures containing the same batch of nanostructures to understand the reproducibility of the measurement. The SERS spectra measured from the five mixtures, as illustrated in Fig. 3d, all clearly show the characteristic Raman peaks of adenine at 734 and 1334 cm^−1^. The average intensities of the peak at 734 cm^−1^ are shown in Fig. 3e, and the relative standard deviation (RSD) of all of the results was as high as 48.4%. The inset of Fig. 3c shows that the Raman laser spot excited only a few nanostructures during a measurement. Therefore, we believe that the high RSD of the results was due to a significant variation in the number of the nanostructures excited by the Raman laser between different measurements. To obtain more reproducible SERS signals, we tested another method of using the synthesized nanostructures for SERS detection of molecules, in which a suspended droplet containing the nanostructures was dried on the surface of a hydrophobic PDMS sheet to accumulate the nanostructures^63,64^. As shown in Fig. 3f, we used the drying process to prepare an area covered by the tightly packed nanostructures to serve as a 3D SERS substrate. While each of the core-satellite nanostructure already had many SERS hot spots at the nanogaps between adjacent satellite nanoparticles, the SERS substrates formed by the accumulation of the nanostructures were able to have even more SERS hot spots because many nanogaps were created between nanostructures. The background SERS spectrum of the substrate is shown in Supplementary Fig. S2. To evaluate the reproducibility of the Raman signals enhanced by the accumulated nanostructures, we prepared five substrates (see Supplementary Fig. S3) similar to that shown in Fig. 3f and then used them to measure SERS spectra of 100 μM adenine. We focused the Raman laser at the central region of each substrate during the measurements. As shown in Fig. 3g,h, the intensities of the peak at 734 cm^−1^ measured using the five substrates were all much stronger than that measured using the dispersed nanostructures, and the RSD of the results measured from the five substrates was only 9.9%. The SERS mapping result shown in Supplementary Fig. S4 also indicates good uniformity of Raman signals. These results clearly show that the core-satellite nanostructures accumulated by the drying process can serve as a sensitive and reproducible SERS substrate. Thus, we used this kind of substrate in the following experiments.

**Figure 4:**
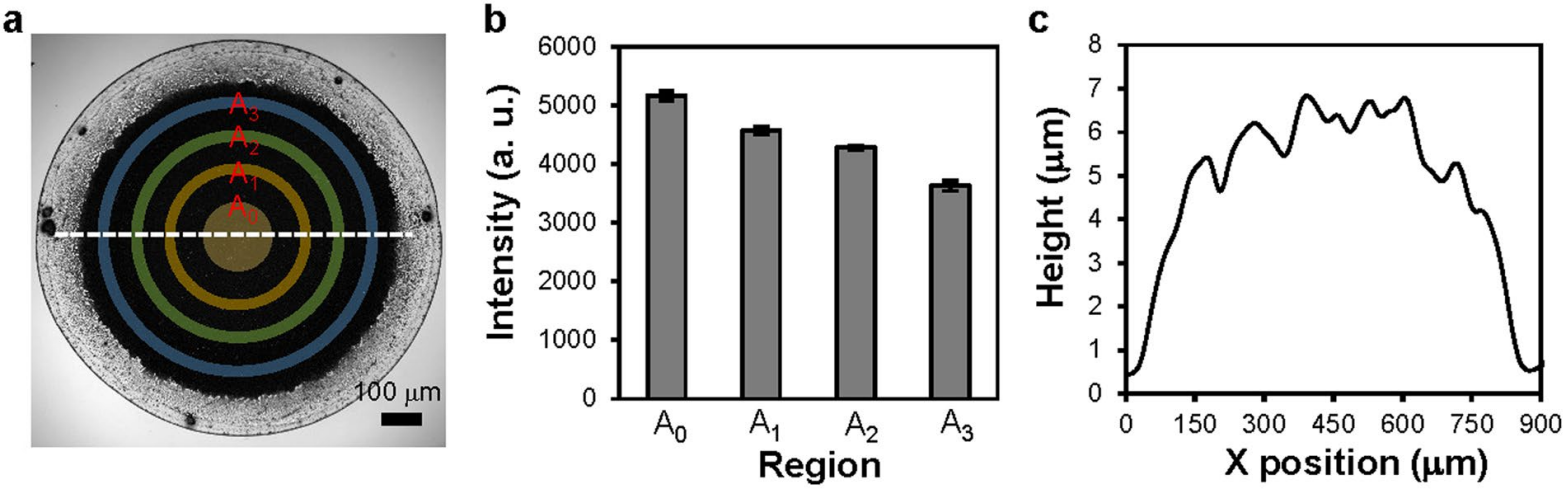
SERS effects at different locations of the substrate and the surface profile of the substrate. (**a**) Representative optical microscope image showing the four regions on the substrate for SERS measurement. (**b**) The intensities of the characteristic Raman peak of adenine at 734 cm^−1^ measured in the four regions shown in (**a**) (*n* = 10 per region). Error bars, SD. (**c**) Surface profile of the substrate measured along the white dashed line in (**a**) using a profiler.

To understand the SERS effects at different locations of the substrate formed by the drying process, we measured SERS spectra of 100 μM adenine in the four regions shown in Fig. 4a. Figure 4b shows that the intensity of the Raman peak of adenine at 734 cm^−1^ was highest in the central region and that the intensity gradually decreased as the location of measurement moved away from the centre of the substrate. In addition, the surface profile of the substrate, which was measured along the white dashed line in Fig. 4a using a surface profiler (KLA-Tencor, P-11), shows that the height of the substrate gradually decreased from the centre to the side, as shown in Fig. 4c. The height was ~6 μm in the central region of the substrate. The results shown in Figs 3c–h and 4a,b demonstrate that the preparation of the substrate was as important as the synthesis of the nanostructures to obtain strong and reproducible Raman signals. Since the droplet that contained the nanostructures was suspended on a hydrophobic PDMS sheet when being dried, no prominent coffee-ring effect was observed on the drying pattern, and the electrostatic adsorption of the nanostructures on the PDMS sheet was not significant either. As a result, a high density of 3D SERS hot spots was created at the centre of the drying pattern, and the Raman enhancement in the central region of the substrate was strong and reproducible when using our drying configuration.

### Optimization of SERS effects

**Figure 5:**
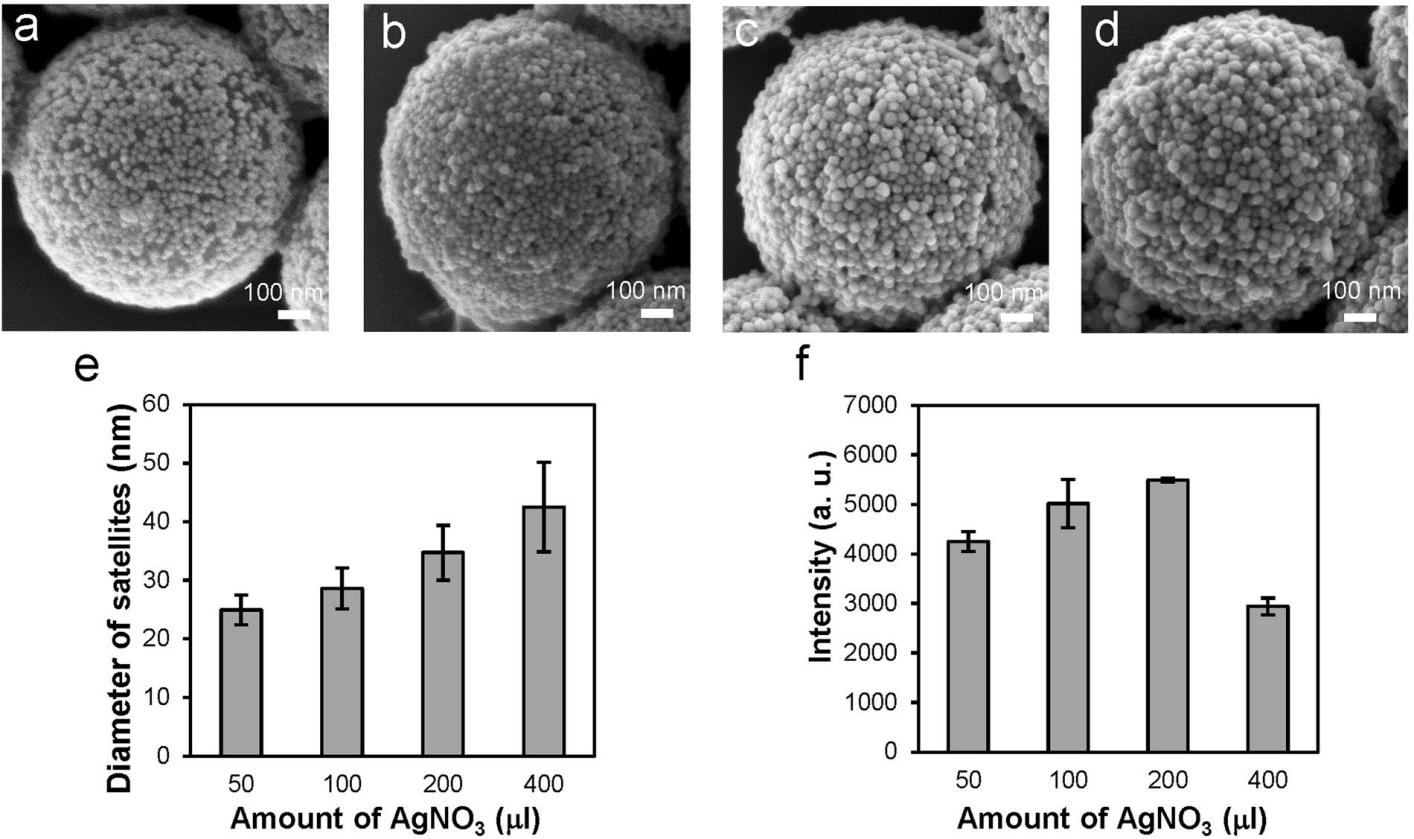
Effects of the thickness of the Ag shells on the geometries and the SERS effects of the core-satellite nanostructures. (**a**–**d**) Representative SEM images showing the nanostructures coated with the Ag shells using (**a**) 50 μl, (**b**) 100 μl, (**c**) 200 μl, and (**d**) 400 μl of 1 mM AgNO_3_. (**e**) The average sizes of the Au-Ag core-shell satellite nanoparticles synthesized using different amounts of AgNO_3_ (*n* = 20 per experimental condition). Error bars, SD. (**f**) The intensities of the characteristic Raman peak of adenine at 734 cm^−1^ measured using the nanostructures that had the satellite nanoparticles of the sizes shown in (**e**) (*n* = 10 per experimental condition). Error bars, SD.

After finding the proper method to utilize the nanostructures for SERS measurements, we optimized the SERS effect of the nanostructure by adjusting the thickness of the Ag shells coated over the satellite nanoparticles. The Ag shell had a major impact on the SERS effect since it affected the interparticle distance and the size of the satellite nanoparticle. During the optimization process, we first tried to grow the Ag shells at three different NaCl concentrations (0.05 M, 0.1 M, and 0.3 M), as it has been reported that the growth of Ag on the surface of a DNA-modified AuNP became more asymmetric as the salt concentration decreased^65^. However, as shown in Supplementary Fig. S5, the NaCl concentration did not cause much difference in the size and shape of the Au-Ag satellite nanoparticles in our study. A possible reason for not seeing prominent effects of the salt concentration was that the densely immobilized AuNPs affected the symmetry of Ag growth and resulted in an asymmetric Ag shell at both high and low salt concentrations, as illustrated in Fig. 3a. In addition to the NaCl concentration, we also tried to vary the amounts of polyvinylpyrrolidone (PVP), (+)-Sodium L-ascorbate (L-SA), and silver nitrate (AgNO_3_) (see Supplementary Table S1), which were the reagents used for Ag growth, while keeping their ratio constant to adjust the thickness the Ag shell. The SEM images of the nanostructures coated with the Ag shells of different thickness (Fig. 5a–d) and the size analysis (Fig. 5e) show that the sizes of the Au-Ag core-shell nanoparticles increased with the amount of AgNO_3_. Nevertheless, the shapes of the Au-Ag nanoparticles became more irregular when the amount of AgNO_3_ was increased above a certain level (Fig. 5d). To evaluate the effects of the Ag shells on SERS measurements, we prepared SERS substrates similar to that shown in Fig. 3f using the nanostructures that had Au-Ag nanoparticles of different sizes (see Fig. 5e) and then utilized the substrates to measure the SERS spectra of 100 μM adenine (see Supplementary Fig. S6). As shown in Fig. 5f, the intensity of the Raman peak at 734 cm^−1^ was highest when the size of the Au-Ag nanoparticle was ~35 nm, which were obtained using 200 μl of 1 mM AgNO_3_. Increasing the size of the nanoparticles from 35 nm to 42 nm did not result in higher Raman enhancement. In fact, the nanostructures with the 42-nm Au-Ag nanoparticles showed the lowest enhancement because some of the nanogaps between the nanoparticles disappeared as the nanoparticles became too large. In addition to the intensity of the Raman peak, we also compared the signal-to-background (S/B) ratio of the Raman peak in the raw SERS spectra before removing the background (see Supplementary Fig. S7). It was found that the S/B ratio was highest for the nanostructures with 35-nm Au-Ag nanoparticles. Based on the results, we used the nanostructures that had 35-nm Au-Ag satellite nanoparticles to measure melamine and NaSCN.

### Detection of melamine and NaSCN

**Figure 6:**
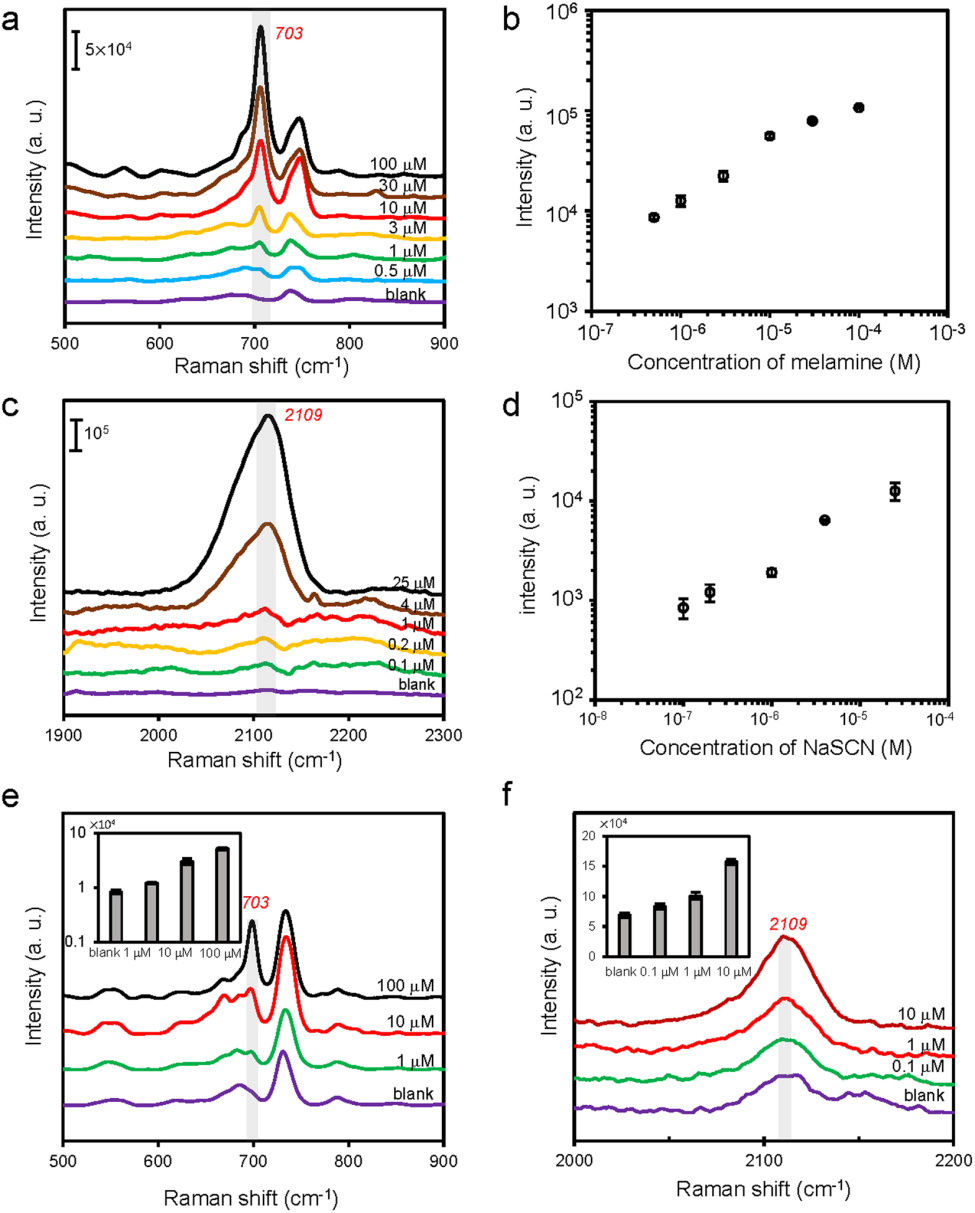
SERS detection of melamine and NaSCN using the synthesized core-satellite nanostructures. (**a**) SERS spectra of melamine in water with concentrations ranging from 0.5 μM to 100 μM. (**b**) A plot of the intensity of the Raman peak at 703 cm^−1^ versus melamine concentration (*n* = 10 per concentration). Error bars, SD. (**c**) SERS spectra of NaSCN in water with concentrations ranging from 0.1 μM to 25 μM. (**d**) A plot of the intensity of the Raman peak at 2109 cm^−1^ versus NaSCN concentration (*n* = 10 per concentration). Error bars, SD. (**e**) SERS spectra of milk samples spiked with different concentrations of melamine (blank, 1 μM, 10 μM, and 100 μM). The inset in (**e**) shows the intensities of the Raman peak at 703 cm^−1^ versus melamine concentration (*n* = 10 per concentration). Error bars, SD. (**f**) SERS spectra of milk samples spiked with different concentrations of NaSCN (blank, 0.1 μM, 1 μM, and 10 μM). The inset in (**f**) shows the intensities of the Raman peak at 2109 cm^−1^ versus NaSCN concentration (*n* = 10 per concentration). Error bars, SD.

We measured SERS spectra of a few different concentrations of melamine and NaSCN, which are both related to the safety of milk, to understand the sensitivity of our SERS substrate. We first measured melamine and NaSCN in water, and then we performed SERS measurements with milk samples spiked with melamine and NaSCN. Melamine, an excess amount of which can cause adverse effects on kidney, had been illegally adulterated in infant formula and animal feeds to increase the apparent protein content because of its high nitrogen content^66^. Unfortunately, contamination of milk products with melamine had resulted in many cases of renal complications and a few cases of death of children in China in 2008^67^. The Maximum Residue Limit (MRL) for melamine has been set to 1 ppm for infant formula and 2.5 ppm for other milk products in the United States and many other countries. Figure 6a shows the Raman spectra of melamine in water measured using the SERS substrates formed by accumulating our core-satellite nanostructures, and Fig. 6b shows the intensities of the characteristic Raman peak of melamine at 703 cm^−1^. As shown in Fig. 6b, we have successfully used our SERS substrates to detect 0.5 μM melamine, which is ~0.06 ppm and is much lower than the MRL.

In addition to melamine, we also measured the Raman spectra of NaSCN in water with concentrations ranging from 0.1 μM to 25 μM (5.8 μg/L-1.45 mg/L of SCN^−^), as shown in Fig. 6c. Thiocyanate (SCN^−^) is an important component of the lactoperoxidase system, which is an effective natural antibacterial system in milk. It has been reported that the average concentration of SCN^−^ in raw milk samples collected from China, New Zealand, and the Netherland was 2.11 mg/kg and that the suggested baseline concentration for SCN^−^ was 9 mg/kg^68^. Low concentration of SCN^−^ in milk does not cause negative health effects, but an excess amount of NaSCN added to milk as a preservative can affect iodine uptake^69^. Figure 6c shows that the characteristic Raman peak of NaSCN at 2109 cm^−1^ was still visible in the spectrum when the concentration of NaSCN was 0.1 μM (5.8 μg/L of SCN^−^), and Fig. 6d shows how the intensity of the Raman peak at 2109 cm^−1^ increased with the concentration.

After measuring melamine and NaSCN in water, we tried to use our SERS substrates to detect melamine and NaSCN in milk. We prepared milk samples spiked with melamine (from 1 μM to 100 μM) and NaSCN (from 0.1 μM to 10 μM) respectively, and then we used the sample preparation procedures detailed in the Materials and Methods section to remove large molecules before SERS measurements. The results, as shown in Fig. 6e,f, indicated that we were able to detect 1 μM (~0.13 ppm) melamine and 0.1 μM NaSCN (5.8 μg/L of SCN^−^) in milk. The detection limit of 1 μM for melamine is already below the MRL for infant formula, and the detection limit of 5.8 μg/L for SCN^−^ is below the baseline concentration of SCN^−^ in raw milk.

## Conclusions

In summary, we synthesized the core-satellite nanostructures that were able to provide strong and reproducible Raman enhancement using a DNA-assisted self-assembly process. Many small gaps between metal nanoparticles were created by densely immobilizing the AuNPs on the polymer beads. To obtain as many gaps as possible, we tested the dependence of the AuNP immobilization on the NaCl concentration and the number ratio of the AuNPs to the polymer beads to maximize the density of the immobilized AuNPs. In addition, since the AuNPs were immobilized on the beads, we were able to reduce the sizes of the gaps between the nanoparticles of the assembled nanostructures by adding a Ag shell to the AuNPs. To optimize the SERS effects, we precisely adjusted the thickness of the Ag shell to make the gaps function as effective SERS hot spots. Moreover, to maximize the number of hot spots under laser irradiation during SERS measurements, we accumulated the synthesized core-satellite nanostructures, which were dispersible in a solution, by drying nanostructure-containing droplets on hydrophobic PDMS sheets. The closely packed nanostructures had densely distributed hot spots on the surfaces of the polymer beads and functioned as sensitive and reproducible 3D SERS substrates for detection of molecules. We successfully used the substrates to detect 0.5 μM melamine and 0.1 μM NaSCN, which are both much lower than their safe levels. Our results demonstrated that this kind of self-assembled SERS substrates could be utilized for sensitive and reproducible detection of molecules related to food safety testing and environmental monitoring applications.

## Materials and Methods

### Materials

All DNA molecules were synthesized by MDBio, Inc. The 20-nm AuNPs and the 1-μm streptavidin-coated polystyrene beads were purchased from Nanocs and Bangs Laboratory, Inc., respectively. Polydimethylsiloxane (PDMS) (Sylgard 184) was purchased from Dow Corning. Other chemical and biochemical products, including sodium chloride (NaCl), sodium phosphate dibasic (Na2HPO4), sodium phosphate monobasic (NaH2PO4¬), sodium dodecyl sulfate (SDS), tris(2-carboxyethyl)phosphine hydrochloride (TCEP-HCl), polyvinylpyrrolidone (PVP) (MW 40,000), (+)-Sodium L-ascorbate (L-SA), silver nitrate (AgNO_3_), Tween 80, phosphate buffer saline (PBS), melamine, sodium thiocyanate (NaSCN) were all obtained from Sigma-Aldrich.

### Synthesis of core-satellite nanostructures

The DNA molecules used in this study were the dual-labelled linker DNA, 5′-thiol-C6-(A)_10_-CAT CCT CAA C-(A)_8_-biotin-3′, and the thiol-labelled A_10_ DNA, 5′-thiol-C6-(A)_10_-3′. The AuNPs were functionalized with the two kinds of thiol-labelled DNA though gold-thiol chemistry using a method similar to that previously reported^62,70^. Briefly, to activate the thiol group of the DNA, we mixed 1.2 μl of 100 μM A_10_ DNA and 1.2 μl of 10 μM linker DNA with 7.6 μl of 5 mM TCEP for 1 h at room temperature. To stabilize the AuNPs for the DNA conjugation process, we mixed 200 μl of the AuNPs with 0.8 μl of 10% (v/v) Tween 80 for 30 min at room temperature, and then we removed unbound Tween 80 by centrifugation at 9000 g for 15 min. After resuspending the AuNPs in 20 μl of water, to functionalize the AuNPs with DNA, we mixed the Tween 80-coated AuNPs with 10 μl of the activated DNA in 10 mM phosphate buffer (pH 7.4) containing 0.1 M NaCl. The mixture was incubated at 55 °C for 5 h, and then the DNA-functionalized AuNPs were washed with 10 mM phosphate buffer (pH 7.4) by centrifugation three times at 9000 g for 15 min. The AuNPs were resuspended in 200 μl of 10 mM phosphate buffer (pH 7.4) after the last centrifugation, and the concentration of NaCl in the phosphate buffer was 0 M, 0.05 M, or 0.3 M when we tested the effects of NaCl concentration on the AuNP immobilization. To immobilize the AuNPs on the streptavidin-coated polymer beads, the DNA-functionalized AuNPs were mixed with a certain amount of the beads for 12 h at room temperature. The volume of the beads mixed with the AuNPs was 1.6 μl, 3.2 μl, or 4.8 μl, which corresponded to the number ratios of 5000:1, 2500:1, and 1667:1 (AuNPs: beads), respectively, when we tested the effects of the number ratio of the AuNPs to the polymer beads on the AuNP immobilization. After immobilizing the AuNPs on the beads to form the core-satellite nanostructures, unbound AuNPs were removed by centrifugation at 6000 g for 3 min, and then the nanostructures were resuspended in 50 μl of 10 mM phosphate buffer (pH 7.4) containing 0.3 M NaCl.

### Growth of Ag shells on AuNPs

We used 1% PVP, 0.1 M L-SA, and 1 mM AgNO_3_ in DI water to add Ag shells to the synthesized core-satellite nanostructures through a chemical reduction method^13,65^. We varied the amounts of PVP, L-SA, and AgNO_3_ used in the synthesis but keeping their ratio constant (see Supplementary Table S1) to adjust the thickness of the Ag shells. We mixed 50 μl of the core-satellite nanostructures with the three reagents in 10 mM phosphate buffer containing 0.3 M NaCl during the synthesis. The mixture was shaken at room temperature for 6 h, and then the nanostructures were washed with water by centrifugation two times at 1000 g for 2 min.

### SERS measurements

We measured SERS spectra using a Raman microscope equipped with a 633 nm HeNe laser (Horiba, LabRAM HR800). The laser was focused by a 20X objective lens with N.A. 0.4 during measurements, and the laser power was 0.8 mW before the objective. We compared two different methods to utilize the nanostructures to enhance Raman signals. When using the first method, we mixed the core-satellite nanostructures with analyte solution (1:1 volume ratio) and then put a drop of the mixture between a microscope slide and a coverslip for SERS measurement. The analytes were measured in solution. When using the second method, a PDMS sheet was first prepared by mixing the silicone elastomer with curing agent (10:1). After the PDMS sheet was cured, we put 3 μl of the synthesized nanostructures on the surface of the PDMS sheet placed on a microscope slide, and then we overturned the microscope slide and the PDMS sheet to dry the droplet. After the hanging droplet was dried completely, we overturned the microscope slide and the PDMS sheet again and then put ~8 μl of analyte solution on the top of the accumulated cores-satellite nanostructures for SERS measurements. The analytes were also measured in solution. Schematic representations of the flow chambers used for SERS measurements are shown in Supplementary Fig. S8.

### Sample preparation procedures for milk samples

Stock solutions of melamine and NaSCN were prepared by dissolving them in DI water. Milk samples were spiked with the analytes by mixing 50 μl of a diluted analyte stock solution and 450 μl of milk. Then, the spiked sample was mixed with1000 μl of methanol and 5 μl of 3.7% HCl for 30 min. Afterwards, large molecules were removed from the milk sample by centrifugation at 20,000 g for 20 min, and then the supernatant was filtered with a 0.22 μm PTFE filter. The filtered solution was then ready for measurement.

## Electronic supplementary material

Supplementary Material

## References

[1] Jones MR, Osberg KD, Macfarlane RJ, Langille MR, Mirkin CA (2011). Templated techniques for the synthesis and assembly of plasmonic nanostructures. Chem. Rev..

[2] Petryayeva E, Krull UJ (2011). Localized surface plasmon resonance: Nanostructures, bioassays and biosensing-A review. Anal. Chim. Acta.

[3] Li JF (2010). Shell-isolated nanoparticle-enhanced Raman spectroscopy. Nature.

[4] Halvorson RA, Vikesland PJ (2010). Surface-enhanced Raman spectroscopy (SERS) for environmental analyses. Environ. Sci. Technol..

[5] Sharma B, Frontiera RR, Henry AI, Ringe E, Van Duyne RP (2012). SERS: Materials, applications, and the future. Mater. Today.

[6] Craig AP, Franca AS, Irudayaraj J (2013). Surface-enhanced Raman spectroscopy applied to food safety. Annu. Rev. Food Sci. Technol..

[7] Luo SC, Sivashanmugan K, Liao JD, Yao CK, Peng HC (2014). Nanofabricated SERS-active substrates for single-molecule to virus detection *in vitro*: A review. Biosens. Bioelectron..

[8] Huang JA, Zhang YL, Ding H, Sun HB (2015). SERS-enabled lab-on-a-chip systems. Adv. Opt. Mater..

[9] Tang X, Dong R, Yang L, Liu J (2015). Fabrication of Au nanorod-coated Fe_3_O_4_ microspheres as SERS substrate for pesticide analysis by near-infrared excitation. J. Raman Spectrosc..

[10] Jiang J, Bosnick K, Maillard M, Brus L (2003). Single molecule Raman spectroscopy at the junctions of large Ag nanocrystals. J. Phys. Chem. B.

[11] Wang H, Levin CS, Halas NJ (2005). Nanosphere arrays with controlled sub-10-nm gaps as surface-enhanced Raman spectroscopy substrates. J. Am. Chem. Soc..

[12] Lim D-K, Jeon K-S, Kim HM, Nam J-M, Suh YD (2010). Nanogap-engineerable Raman-active nanodumbbells for single-molecule detection. Nat. Mater..

[13] Lee J-H (2012). Tuning and maximizing the single-molecule surface-enhanced Raman scattering from DNA-tethered nanodumbbells. ACS Nano.

[14] Zhang Z, Zhang S, Lin M (2014). DNA-embedded Au-Ag core-shell nanoparticles assembled on silicon slides as a reliable SERS substrate. Analyst.

[15] Tan SJ, Campolongo MJ, Luo D, Cheng W (2011). Building plasmonic nanostructures withDNA. Nat. Nanotechnol..

[16] Xing H (2012). DNA-directed assembly of asymmetric nanoclusters using Janus nanoparticles. ACS Nano.

[17] Kumar A, Hwang JH, Kumar S, Nam JM (2013). Tuning and assembling metal nanostructures with DNA. Chem. Commun..

[18] Rothemund PWK (2006). Folding DNA to create nanoscale shapes and patterns. Nature.

[19] Park SY (2008). DNA-programmable nanoparticle crystallization. Nature.

[20] Hill HD (2008). Controlling the lattice parameters of gold nanoparticle FCC crystals with duplex DNA linkers. Nano Lett..

[21] Castro CE (2011). A primer to scaffolded DNA origami. Nat. Methods.

[22] Wei B, Dai MJ, Yin P (2012). Complex shapes self-assembled from single-stranded DNA tiles. Nature.

[23] Wang ZG, Ding BQ (2014). Engineering DNA self-assemblies as templates for functional nanostructures. Accounts Chem. Res..

[24] Xu X, Rosi NL, Wang Y, Huo F, Mirkin CA (2006). Asymmetric functionalization of gold nanoparticles with oligonucleotides. J. Am. Chem. Soc..

[25] Huo F, Lytton‐Jean AK, Mirkin CA (2006). Asymmetric functionalization of nanoparticles based on thermally addressable DNA interconnects. Adv. Mater..

[26] Fan JA (2011). DNA-enabled self-assembly of plasmonic nanoclusters. Nano Lett..

[27] Zheng Y (2013). DNA-directed self-assembly of core-satellite plasmonic nanostructures: a highly sensitive and reproducible near-IR SERS sensor. Adv. Funct. Mater..

[28] Zhao Y (2015). Double Detection of Mycotoxins Based on SERS Labels Embedded Ag@Au Core-Shell Nanoparticles. ACS Appl. Mater. Interfaces.

[29] Yoon JH, Lim J, Yoon S (2012). Controlled assembly and plasmonic properties of asymmetric core-satellite nanoassemblies. ACS Nano.

[30] Gandra N, Abbas A, Tian L, Singamaneni S (2012). Plasmonic planet-satellite analogues: hierarchical self-assembly of gold nanostructures. Nano Lett..

[31] Xie W, Walkenfort B, Schlücker S (2012). Label-free SERS monitoring of chemical reactions catalyzed by small gold nanoparticles using 3D plasmonic superstructures. J. Am. Chem. Soc..

[32] Yoon JH, Yoon S (2013). Probing interfacial interactions using core-satellite plasmon rulers. Langmuir.

[33] Chang H (2014). Ag shell-Au satellite hetero-nanostructure for ultra-sensitive, reproducible, and homogeneous NIR SERS activity. ACS Appl. Mater. Interfaces.

[34] Rong Z, Xiao R, Wang C, Wang D, Wang S (2015). Plasmonic Ag core-satellite nanostructures with a tunable silica-spaced nanogap for surface-enhanced Raman scattering. Langmuir.

[35] Kim NH, Lee SJ, Moskovits M (2011). Reversible tuning of SERS hot spots with aptamers. Adv. Mater..

[36] Ma W (2014). Ultrasensitive aptamer-based SERS detection of PSAs by heterogeneous satellite nanoassemblies. Chem. Commun..

[37] Feng J (2015). A SERS active bimetallic core-satellite nanostructure for the ultrasensitive detection of Mucin-1. Chem. Commun..

[38] Stoerzinger KA, Lin JY, Odom TW (2011). Nanoparticle SERS substrates with 3D Raman-active volumes. Chem. Sci..

[39] Zhang Q, Lee YH, Phang IY, Lee CK, Ling XY (2014). Hierarchical 3D SERS substrates fabricated by integrating photolithographic microstructures and self-assembly of silver nanoparticles. Small.

[40] Sun YD (2015). Three-dimensional hotspots in evaporating nanoparticle sols for ultrahigh Raman scattering: solid-liquid interface effects. Nanoscale.

[41] Liu HL, Yang LB, Liu JH (2016). Three-dimensional SERS hot spots for chemical sensing: Towards developing a practical analyzer. Trac-Trends Anal. Chem..

[42] Mühlig S (2011). Self-assembled plasmonic core–shell clusters with an isotropic magnetic dipole response in the visible range. ACS Nano.

[43] Choi I (2012). Core-satellites assembly of silver nanoparticles on a single gold nanoparticle via metal ion-mediated complex. J. Am. Chem. Soc..

[44] Yoon JH, Zhou Y, Blaber MG, Schatz GC, Yoon S (2013). Surface plasmon coupling of compositionally heterogeneous core–satellite nanoassemblies. J. Phys. Chem. Lett..

[45] Indrasekara ASD, Thomas R, Fabris L (2015). Plasmonic properties of regiospecific core-satellite assemblies of gold nanostars and nanospheres. Phys. Chem. Chem. Phys..

[46] Zhang T (2015). Construction of plasmonic core-satellite nanostructures on substrates based on DNA-directed self-assembly as a sensitive and reproducible biosensor. ACS Appl. Mater. Interfaces.

[47] Höller RP (2016). Protein-assisted assembly of modular 3D plasmonic raspberry-like core/satellite nanoclusters: correlation of structure and optical properties. ACS Nano.

[48] Gandra N, Singamaneni S (2012). “Clicked” plasmonic core–satellites: covalently assembled gold nanoparticles. Chem. Commun..

[49] Ruan Q, Shao L, Shu Y, Wang J, Wu H (2014). Growth of monodisperse gold nanospheres with diameters from 20 nm to 220 nm and their core/satellite nanostructures. Adv. Opt. Mater..

[50] Yang N (2017). An ultrasensitive near-infrared satellite SERS sensor: DNA self-assembled gold nanorod/nanospheres structure. RSC Adv..

[51] Zhu Y (2015). A poly adenine-mediated assembly strategy for designing surface-enhanced resonance Raman scattering substrates in controllable manners. Anal. Chem..

[52] Li A (2016). A SERS-active sensor based on heterogeneous gold nanostar core–silver nanoparticle satellite assemblies for ultrasensitive detection of aflatoxin B1. Nanoscale.

[53] Yang K (2017). A novel SERS-based magnetic aptasensor for prostate specific antigen assay with high sensitivity. Biosens. Bioelectron..

[54] Tsoutsi D (2011). Quantitative surface-enhanced Raman scattering ultradetection of atomic inorganic ions: the case of chloride. ACS Nano.

[55] Ahijado-Guzmán R, Gómez-Puertas P, Alvarez-Puebla RA, Rivas G, Liz-Marzán LM (2012). Surface-enhanced Raman scattering-based detection of the interactions between the essential cell division FtsZ protein and bacterial membrane elements. ACS Nano.

[56] Sun Z, Du J, Lv B, Jing C (2016). Satellite Fe_3_O_4_@SiO_2_-Au SERS probe for trace Hg^2+^ detection. RSC Adv..

[57] He R (2014). Plasmonic core/satellite heterostructure with hierarchical nanogaps for Raman spectroscopy enhanced by shell‐isolated nanoparticles. Adv. Opt. Mater..

[58] Ingelfinger JR (2008). Melamine and the global implications of food contamination. N. Engl. J. Med..

[59] Xin H, Stone R (2008). Tainted milk scandal: Chinese probe unmasks high-tech adulteration with melamin. Science.

[60] Haddadin MS, Ibrahim SA, Robinson RK (1996). Preservation of raw milk by activation of the natural lactoperoxidase systems. Food Control.

[61] Reiter B, Harnulv G (1984). Lactoperoxidase antibacterial system - natural occurrence, biological functions and practical applications. J. Food Prot..

[62] Xu SM, Yuan H, Xu A, Wang J, Wu LJ (2011). Rapid synthesis of stable and functional conjugates of DNA/gold nanoparticles mediated by Tween 80. Langmuir.

[63] Avci E, Culha M (2013). Influence of droplet drying configuration on surface-enhanced Raman scattering performance. RSC Adv..

[64] Wang HY (2015). A hanging plasmonic droplet: three-dimensional SERS hotspots for a highly sensitive multiplex detection of amino acids. Analyst.

[65] Lee JH, Kim GH, Nam JM (2012). Directional synthesis and assembly of bimetallic nanosnowmen with DNA. J. Am. Chem. Soc..

[66] Chan EYY, Griffiths SM, Chan CW (2008). Public-health risks of melamine in milk products. Lancet.

[67] Xiu CB, Klein KK (2010). Melamine in milk products in China: Examining the factors that led to deliberate use of the contaminant. Food Policy.

[68] Yong L (2017). Investigation of concentration of thiocyanate ion in raw cow’s milk from China, New Zealand and the Netherlands. Food Chem..

[69] Tonacchera M (2004). Relative potencies and additivity of perchlorate, thiocyanate, nitrate, and iodide on the inhibition of radioactive iodide uptake by the human sodium iodide symporter. Thyroid.

[70] Yu LH, Chen YF (2015). Concentration-dependent thermophoretic accumulation for the detection of DNA using DNA-functionalized nanoparticles. Anal. Chem..

