# Supplementary material for: Three-Dimensional SERS Substrates Formed with Plasmonic Core-Satellite Nanostructures
Source: Sci Rep. 2017 Oct 12;7:13066. doi: 10.1038/s41598-017-13577-9 (PMC5638830; doi:10.1038/s41598-017-13577-9)
Supplement: Supplementary file 1 — Supplementary Material [file 41598_2017_13577_MOESM1_ESM.pdf]

Supplementary Material

Three-Dimensional SERS Substrates Formed with Plasmonic Core-Satellite Nanostructures

Li-An Wu[1], Wei-En Li[1], Ding-Zheng Lin[2] & Yih-Fan Chen[1, 3, *]

[1]Institute of Biophotonics, National Yang-Ming University, Taipei 112, Taiwan

[2]Material and Chemical Research Laboratory, Industrial Technology Research Institute, Hsinchu 310, Taiwan

[3]Biophotonics and Molecular Imaging Research Center, National Yang-Ming University, Taipei 112, Taiwan

*Y.F.C. (email: chenyf@ym.edu.tw)

Extinction spectra of the core-satellite nanostructures

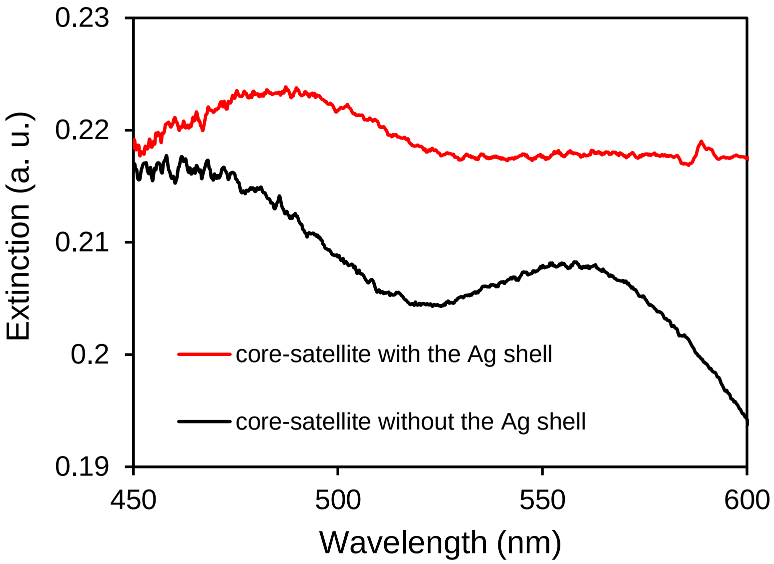

Figure S1. Extinction spectra of the core-satellite nanostructures in water before (black line) and after (red line) the addition of the Ag shell. The sizes of the satellite nanoparticles were ~32 nm after the addition of the Ag shell.

Background SERS spectrum of the substrate

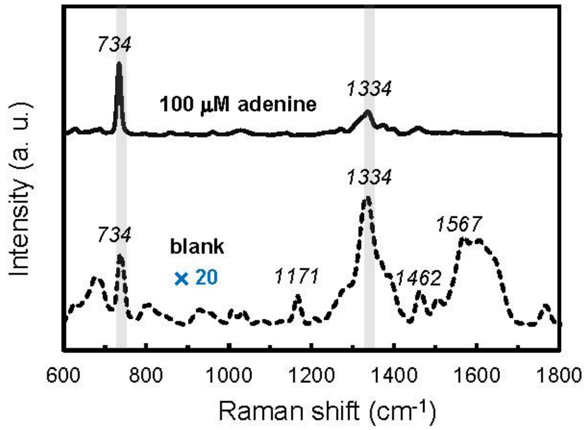

Figure S2. Raman signals of DNA, streptavidin, and polystyrene in the background SERS spectrum. SERS spectrum of 100 µM adenine (solid line) and background SERS spectrum (dashed line) measured using the accumulated core-satellite nanostructures as SERS substrates. The background SERS spectrum (dashed line) is multiplied by a factor of 20 for clarity. The characteristic Raman peaks of adenine at 734 and 1334 cm^{-1} and the peaks of streptavidin-biotin complexes[1] at 1171, 1462, and 1567 cm^{-1} are visible in the background SERS spectrum. The peak at ~1600 cm^{-1}, which could be from polystyrene[2], is also visible in the spectrum.

Reproducibility on the accumulation of the core-satellite nanostructures

We prepared five SERS substrates using the drying process and then utilized them to measure SERS spectra of 100 μM adenine. The SERS spectra are shown in Figure 3g.

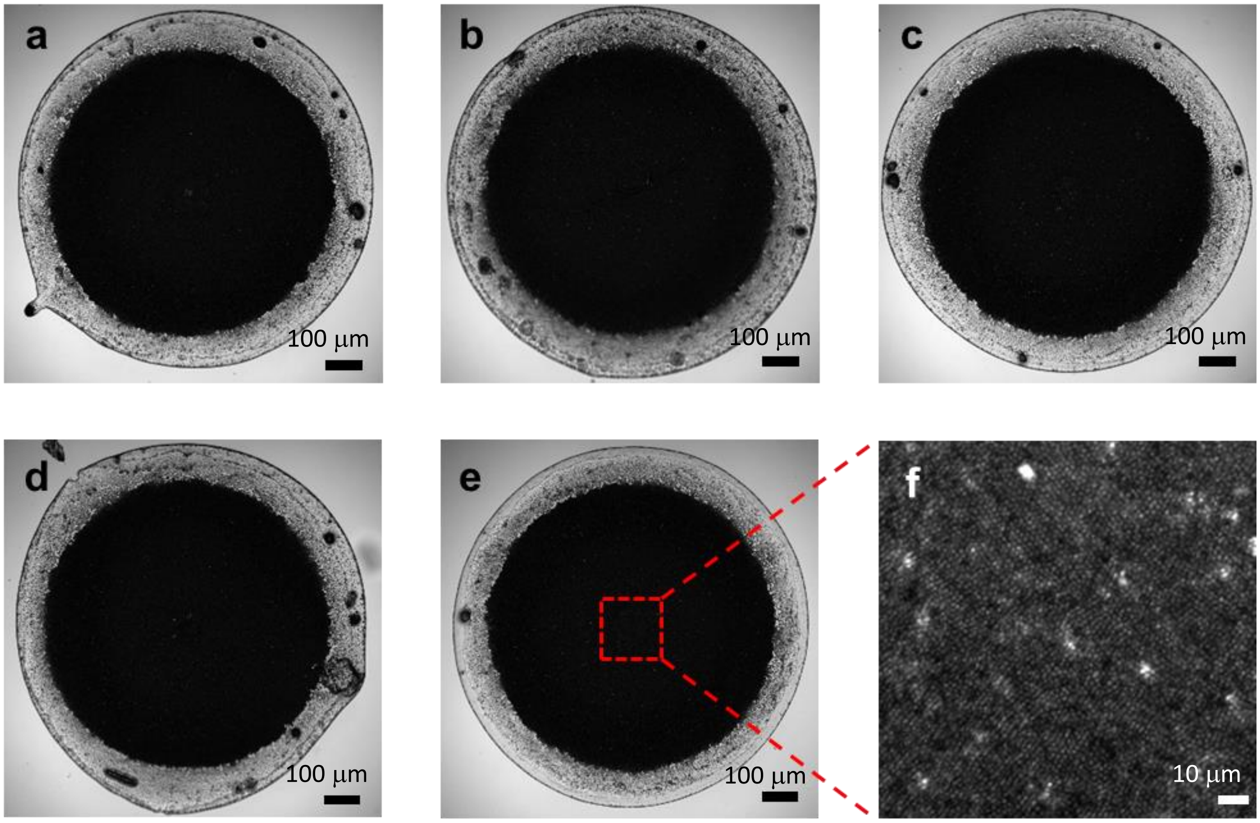

Figure S3. SERS substrates formed by drying nanostructure-containing droplets suspended on PDMS sheets. (a-e) Representative optical microscope images showing the five substrates used to measure the SERS spectra shown in Figure 3g. (f) Zoom-in image of the area marked with a red square in (e).

SERS mapping of the substrates

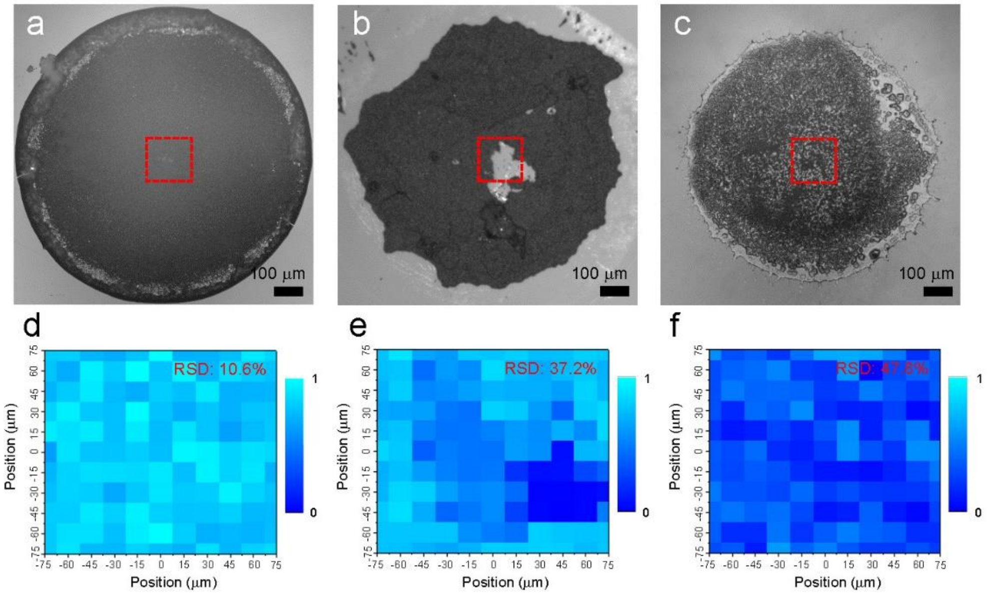

Figure S4. Effects of the density of the nanostructures on SERS. (a-c) SERS substrates prepared by the drying process using (a) 100%, (b) 50%, and (c) 20% of the original amount of the nanostructures (see Materials and Methods). The red dashed squares indicate the area where the SERS mapping measurements were performed. The SERS mapping region is within the central region shown in Fig. 4a. (d-f) SERS mapping of the substrates shown in (a), (b), and (c) using the 734 cm^{-1} peak of 100 μM adenine solution. The intensities of the Raman peak at 734 cm^{-1} measured using the three substrates were all normalized by the highest intensity obtained with the substrate shown in (a).

Effects of NaCl concentration on the growth of the Ag shells

We synthesized the Ag shells at three different NaCl concentrations (0.05 M, 0.1 M, and 0.3 M). As shown in Figure S5a-c, the NaCl concentration did not cause much difference in the size and shape of the Au-Ag satellite nanoparticles in our study.

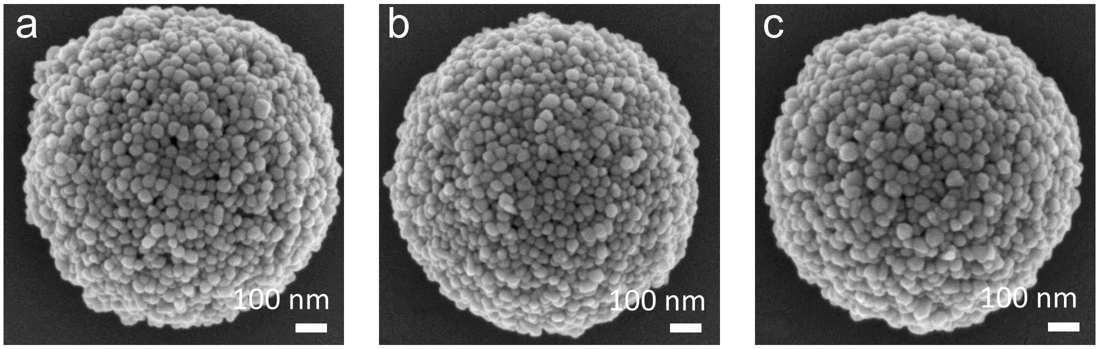

Figure S5. Effects of the NaCl concentration on the growth of the Ag shells. (a-c) Representative SEM images showing the nanostructures coated with the Ag shells at (a) 0.05 M, (b) 0.1 M, and (c) 0.3 M NaCl solution.

Amount of the reagents used to grow Ag shells on AuNPs

To adjust the thickness of the Ag shell on the AuNPs, we varied the amounts of PVP, L-SA, and $AgNO_3$ used in the synthesis, as shown in Table S1.

Table S1. The amounts of the reagents used to form the Ag shells around the AuNPs.

Unit: μl

	1X	2X	4X	8X
**1% PVP**	16.7	33.3	66.6	133.2
**0.1 M L-SA**	50	100	200	400
**1 mM AgNO3**	50	100	200	400

Effects of the thickness of the Ag shells on the SERS effects

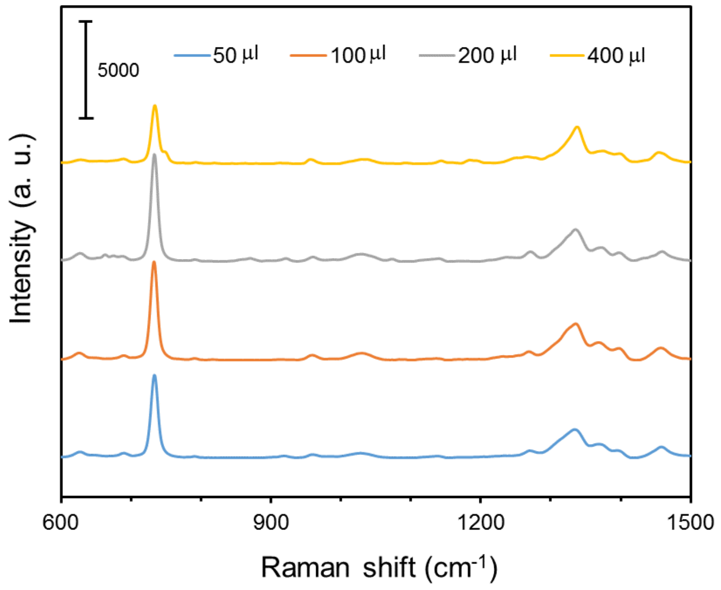

Figure S6. SERS spectra of 100 µM adenine measured using the nanostructures that were coated with the Ag shells using different volumes of 1 mM $AgNO_3$. The intensities of the characteristic Raman peak of adenine at 734 cm^{-1} are shown in Figure 4f.

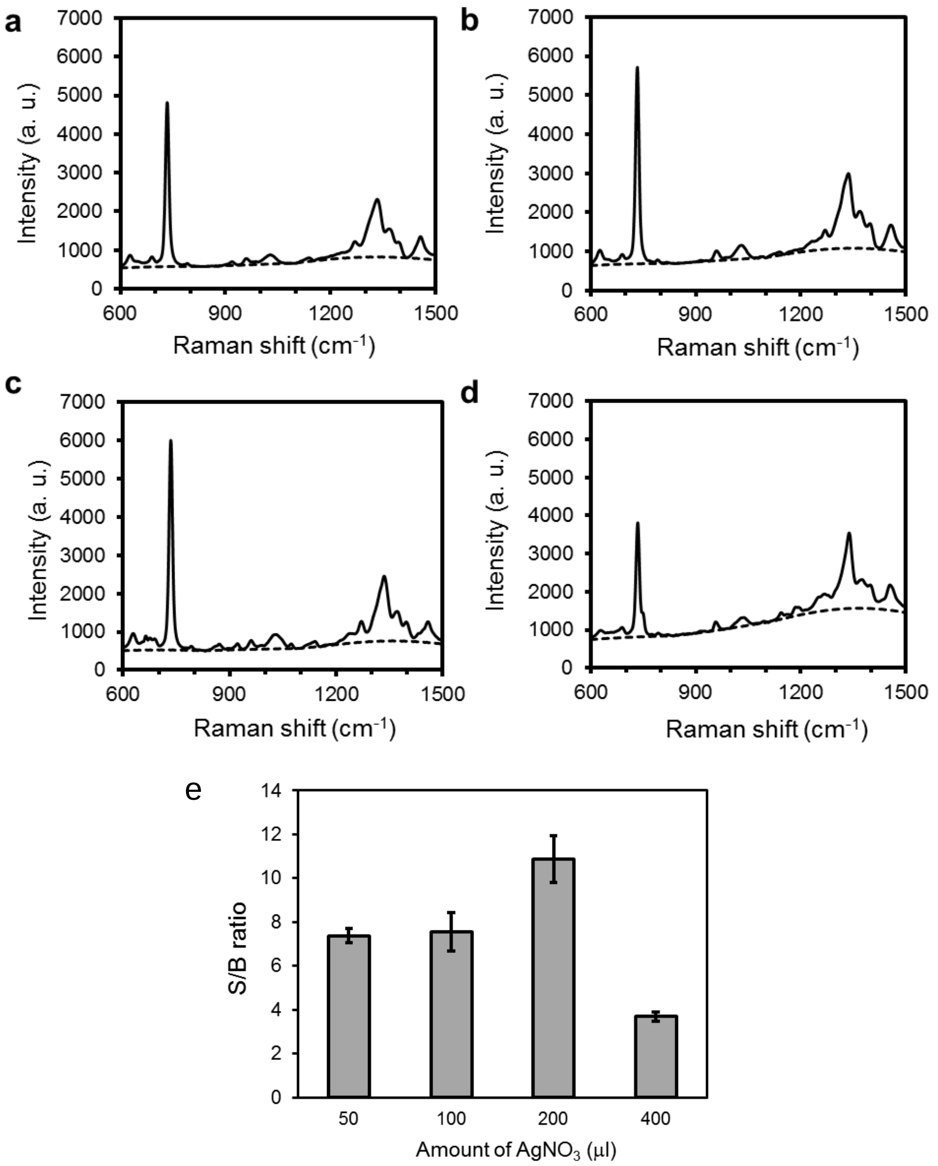

Figure S7. Effects of the thickness of the Ag shells on the signal-to-background (S/B) ratio of the Raman peak. (a-d) Raw SERS spectra (solid lines) of 100 μM adenine measured using the nanostructures that were coated with the Ag shells using (a) 50 μl, (b) 100 μl, (c) 200 μl, and (d) 400 μl of 1 mM $AgNO_3$. The dashed lines indicate the backgrounds. (e) The S/B ratio of the Raman peak at 734 cm^{-1} in the raw SERS spectra shown in (a-d) (n = 10 per experimental condition). Error bars, SD.

Flow chambers used for SERS measurements

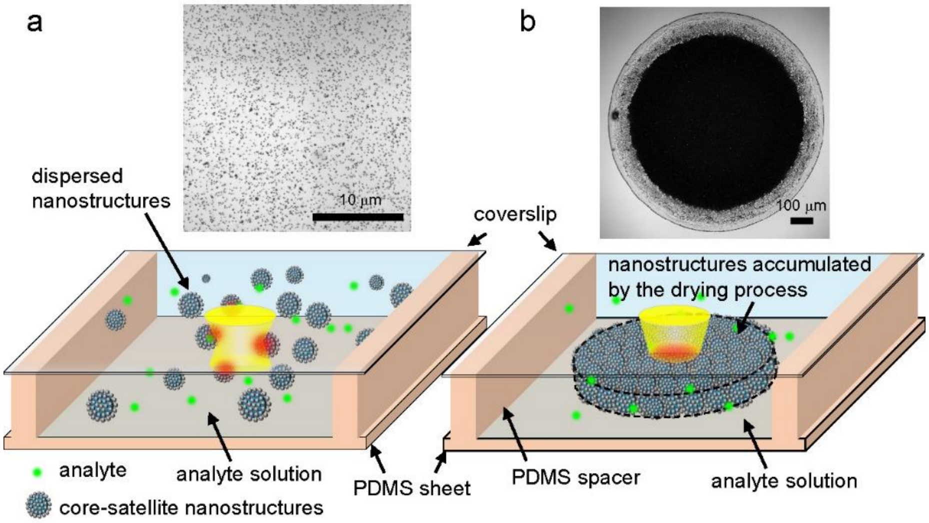

Figure S8. Schematic representations of the flow chambers used for SERS measurements. (a) The analytes were mixed with the nanostructures and were measured in solution. (b) The nanostructures were accumulated by the drying process to form the SERS substrate, and then the analyte solution was injected in the flow chamber that had the SERS substrate for SERS measurements.

Storage stability of the core-satellite nanostructures

The core-satellite nanostructures were stored in TE buffer at 4°C. To understand the storage stability of the nanostructures, we used the nanostructures that had been stored for different periods to measure SERS spectra of 100 μM adenine.

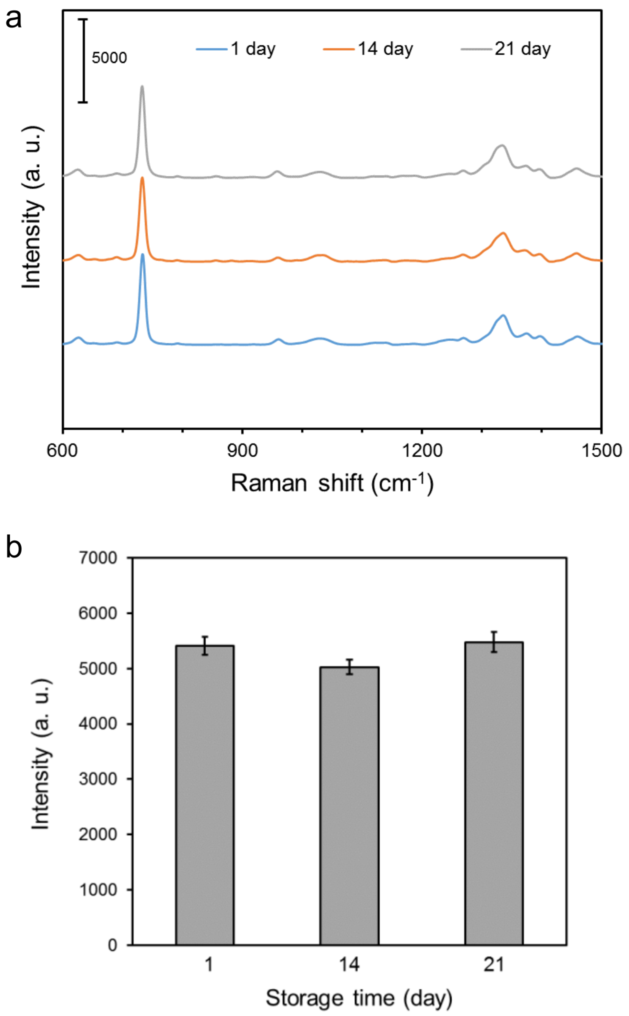

Figure S9. Storage stability of the core-satellite nanostructures. (a) SERS spectra and (b) the intensities of the characteristic Raman peak of adenine at 734 cm^{-1} obtained using the nanostructures that had been stored for different periods.

References

1 Wang, H. & Schultz, Z. D. The chemical origin of enhanced signals from tip-enhanced Raman detection of functionalized nanoparticles. *Analyst* **138**, 3150-3157 (2013).

2 Anema, J. R., Brolo, A. G., Felten, A. & Bittencourt, C. Surface-enhanced Raman scattering from polystyrene on gold clusters. *J. Raman Spectrosc.* **41**, 745-751 (2010).
